# Persecution

**Published:** 1876-12

**Authors:** 


### FAMOUS THROUGH PERSECUTION.

Dr. E. B. Foote, a very entertaining and popular writer, and a scientific gentleman, withal, has received about one hundred thousand dollars of advertising through the efforts of a Mr. Comstock, who complained that the Doctor was employing the United States mails for the conveyance of immodest literature, against which offence a clumsy sort of a law exists. So the Doctor got fined, and subsequently published a voluminous account of his trial for the technical offence. The absurdity of the enactment was made clear, so far as it has been construed as having any reference to medical works, and a few more cases of this sort would be quite likely to breed a good sized rebellion among medical writers and publishers. Of course there must be a scape-goat of some kind, so the animus of the prosecution is laid by some upon the express companies, who seek to exclude books of large circulation from the mails in order that they may monopolize the carrying trade.

The Doctors Foote—father and son—publish a very able magazine for general reading, called *Dr. Foote's Health Monthly*, and in this work the whole prosecution and persecution business has been reported, together with hosts of letters from the public at large expressive of sympathy, and contributing money for the payment of the fine. This money is accepted, and credited up to the sender on account of subscriptions at $1 a year to the *Health Monthly*.

As for the character of Dr. Foote's books, we can only say that while they tell a good deal of naked truth in plain language, he must be a prurient prude indeed, who is able to detect anything immodest in the expressions made use of. Abuses and errors are pointed out in vigorous language, but there is no indelicacy of utterance, and the mind which could thus construe it, must be polluted indeed. As medical writers, we protest against the law not less than against this last construction of it, and shall see to it that it be so modified by Congress as to exclude medical men from its operation, since they necessarily have to handle topics which the world has been taught to consider "delicate." Corrupters of the morals of the young, who infest our cities and work secret pollution through obscene literature and pictures among the inexperienced, should be swept from the earth as vile enemies of mankind ; but we can not long permit such misguided censorship of press or mails as is calculated to lessen scientific discussion, or keep the world in ignorance of vital truth.

---

SMALLPOX.—"I am willing to risk my reputation as a public man," wrote Edward Hine to the Liverpool *Mercury*, "if the worst case of smallpox cannot be cured in three days, simply by the use of cream of tartar. One ounce of cream of tartar dissolved in a pint of water, drank at intervals, when cold, is a certain, never-failing remedy. It has cured thousands, never leaves a mark, never causes blindness, and avoids tedious lingerings."

